# The Rise and Fall of Basic Science

**DOI:** 10.3389/fnsys.2015.00160

**Published:** 2015-11-20

**Authors:** Ehud Ahissar

**Affiliations:** The Department of Neurobiology, Weizmann Institute of ScienceRehovot, Israel

**Keywords:** politics of science, flagship projects, the human brain project, chemotaxis, novotaxis

When you don't have a clue where to look for something you are interested in, such as cookies hidden in the kitchen, the best strategy may be a random search. If that something can be remotely sensed by you, e.g., smelled, then your search would be facilitated by adding gradient-sensing—if the gradient over two or more samples taken along a path is positive don't change the path, if not—select another path (randomly). This algorithm has been adapted by microbes while searching for food (known as chemotaxis)—it ensures that the microbes, as a community, would find any reachable food located in their vicinity. The same algorithm appears to have subserved scientific search for several centuries with a great success.

Scientists typically direct their basic research according to internal drives that are modulated by the scientific community. The preservation of independence of research among individual scientists allows a wide coverage of the search field that is available to human research at a given time. Mechanisms that are based on peer review provide (ideally, although not always practically) the required gradient sensing component—research paths that yield positive gradients are supported and followed by others, while paths that do not yield positive gradients are abandoned (Figure 1). Evolution tells us that such a “novotaxis” mechanism is the best mechanism for revealing novel information that lie out there at our reach.

**Figure 1:**
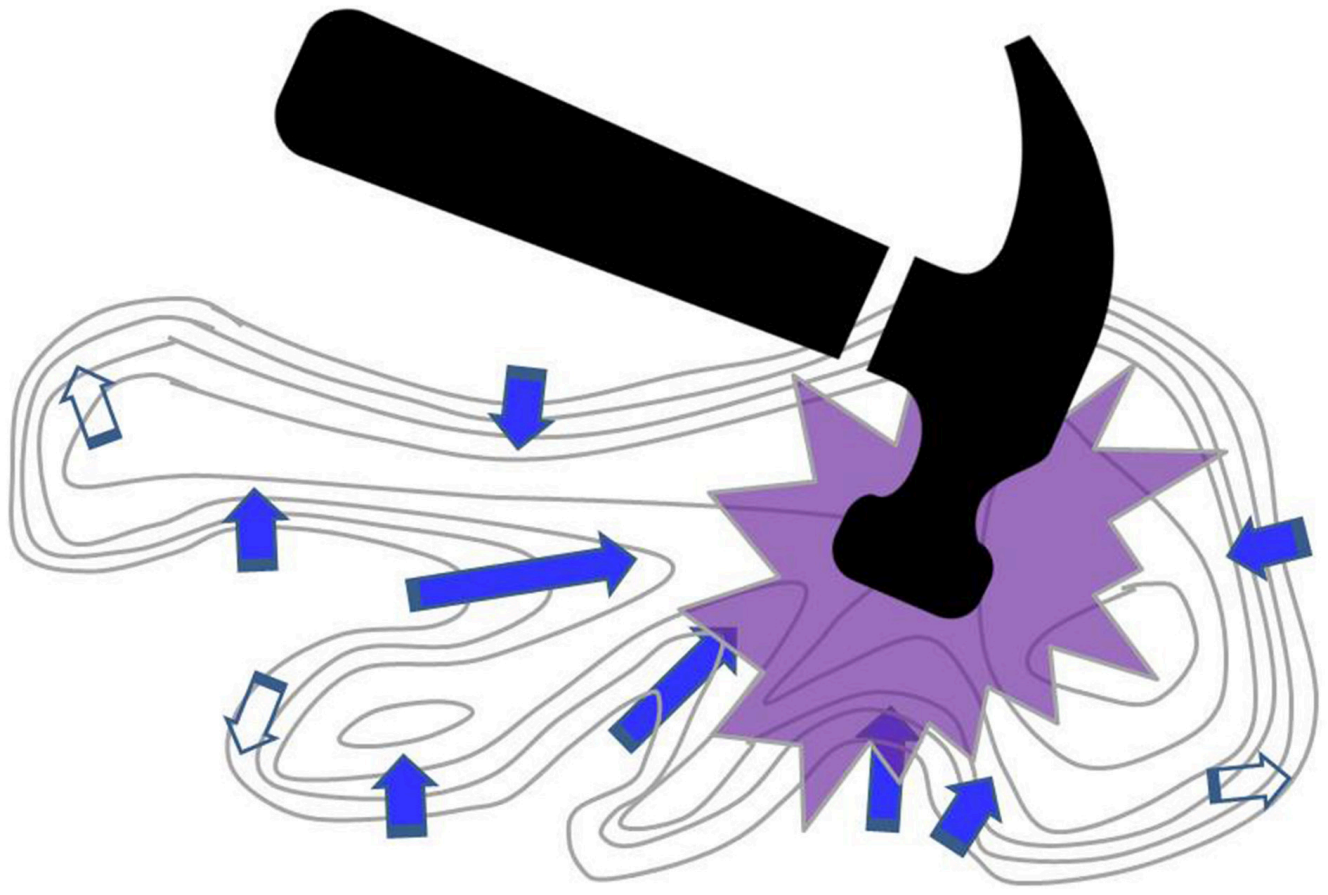
**A topographic metaphor**. In basic science, individual investigators (arrows) follow their intuition, knowledge, and creative thinking along fine-grained gradients, converging as a group toward the underlying principles of nature—the centers of the contours. Public funding should aim to recognize those lines of research that promise to follow positive gradients (filled arrows). Disproportionate top-down funding initiatives like the European Human Brain Project flagship, symbolized by the massive hammer, fail to recognize the value of individual research lines and threaten, upon impact, to destroy the existing research fabric.

The general x-taxis (chemo/photo/novo/… -taxis) mechanism appears to be preserved along evolution as the default exploration mechanism employed when facing novel frontiers. This general mechanism is repeatedly replaced, along evolution, by more direct mechanisms implementing faster and more efficient, but also costly, algorithms. Imagine for example an evolutionary path leading from chemotaxis to object localization. Mechanisms of direct localization of objects, such as electrolocation in electric fish, echolocation in bats or visual localization in primates were stabilized along evolution as they increase the survivability of the individual by allowing a quick localization of food. Interestingly, however, the x-taxic mechanism did not disappear during this evolutionary process, but has rather been rendered to frontiers that are beyond immediate reach. For example, finding the next hunting zone. In other words, x-taxis remains the default search mechanism at the frontiers of knowledge, while tasks for which enough knowledge has been accumulated are implemented with directed mechanisms. Basic science forms a modern expression of this principle, with its independent search projects and a peer-review based gradient sensing mechanisms. However, this front, which has been exploited impressively during the last few centuries, seems to face a dramatic challenge, which may signal its falling phase. The challenge is what may be called the politics of science.

During the last years there is a trend that has gained increasing popularity among funding authorities. The trend is characterized by replacing independent scientific research with top-down directed focused search. Importantly, this trend started with projects that do not affect basic science, but it continues with projects that seriously threaten basic science. Among the first precursors of this process were the human genome project at the US and the particle accelerator in Europe. In these projects the basic scientific principles (e.g., genetic codes and physical laws) were known, the path for scientific findings was clear and the only missing component was an intensive application of measurements. This can be compared to the evolutionary path leading from chemotaxis to object localization; you know that the food is out there, you know how it feels and thus you can accelerate its localization by applying intensive measurements using high spatiotemporal resolution. However, the most salient recent example in this trend, the EC Human Brain Project “flagship” project, is totally out of this alley. In this case no principle (e.g., neural codes, transformation laws) is known and no scientific path has proven to be the right path to the target—understanding the brain. Thus, selecting a (any) single direction here has roughly zero chance to hit the target.

Thus, while “flagship” projects focusing on industrial directions make sense, there is no sense in similar projects aiming at basic science. On the contrary, it goes against the paradigm that proved to be successful during the last several centuries. The impressive progress in neuroscience, resulting from the accumulated gains of the full set of individual researchers, could thus be at risk.

## Conflict of interest statement

The author declares that the research was conducted in the absence of any commercial or financial relationships that could be construed as a potential conflict of interest.

